# A Rare Case of Aortoatrial Fistula from Streptococcal Endocarditis

**DOI:** 10.1155/2017/8189658

**Published:** 2017-07-05

**Authors:** Hammad Arshad, Meilin Young, Parth Rali

**Affiliations:** ^1^Division of Pulmonary and Critical Care, Allegheny General Hospital, Pittsburgh, PA 15212, USA; ^2^Division of Internal Medicine, Allegheny General Hospital, Pittsburgh, PA 15212, USA

## Abstract

We represent an unfortunate case of postinfluenza streptococcal endocarditis in a 34-year-old healthy male. He presented with hypoxic respiratory failure and was found to have mitral and aortic valve vegetation. Hospital course was complicated by the presence of an aortoatrial fistula from an aortic root abscess, persistent septic shock, and multiorgan failure.

## 1. Case Presentation

A 34-year-old male patient with a past medical history of asthma presented as a transfer for new onset hypoxemic respiratory failure and management of pericardial effusion. The patient recently developed weakness, fatigue, and shortness of breath and presented to his primary care physician's office for evaluation. The patient tested positive for H1N1 influenza A and was treated with oseltamivir.

**Figure 1:**
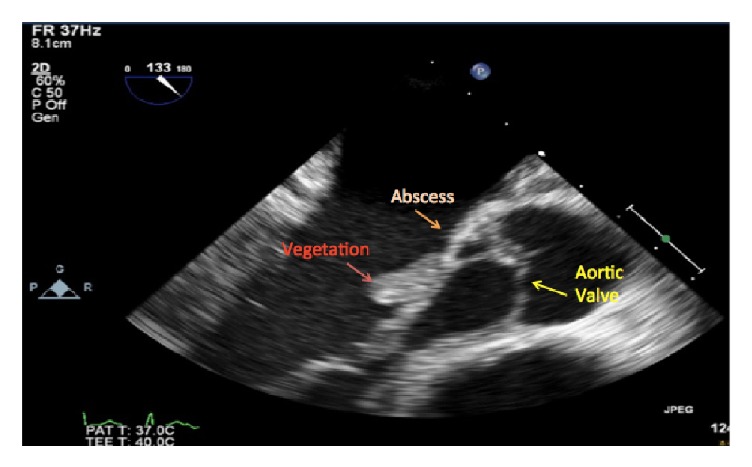
Valvular vegetation with possible abscess.

**Figure 2:**
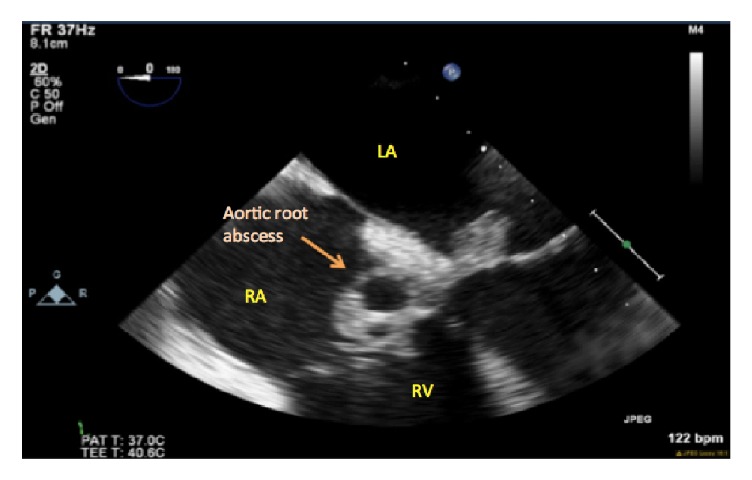
Aortic root mass with central clearing, indicating an abscess.

**Figure 3:**
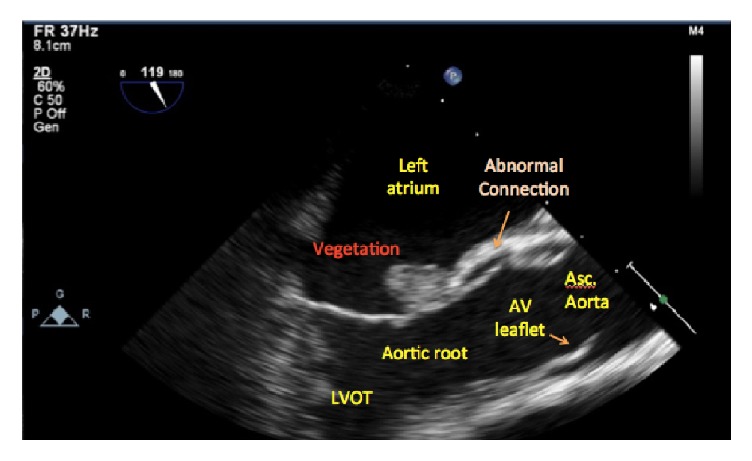
Concern for presence of an abnormal tract from the aorta.

**Figure 4:**
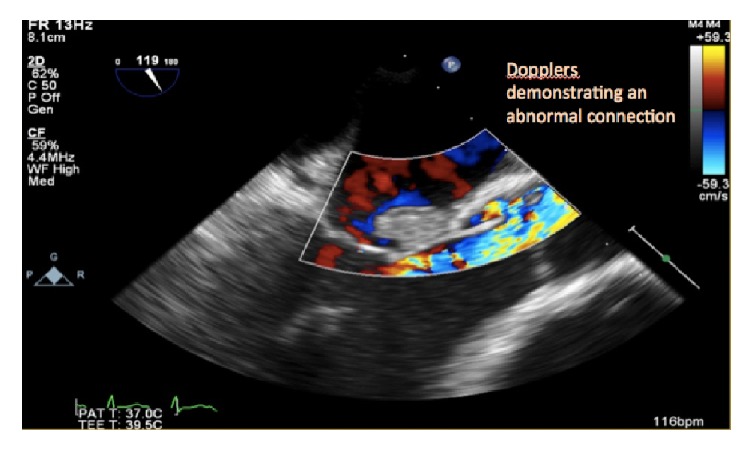
Doppler confirming an abnormal flow tract from the aorta.

**Figure 5:**
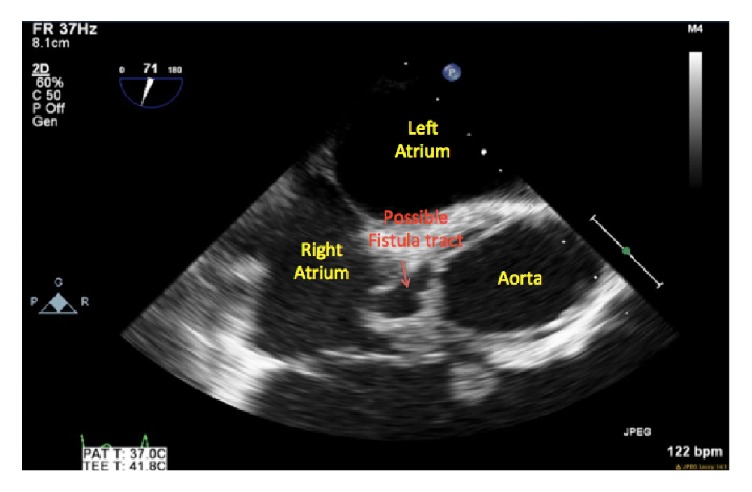
Axial image of the aortic root abscess demonstrating a possible fistula between the aorta and the right atrium.

**Figure 6:**
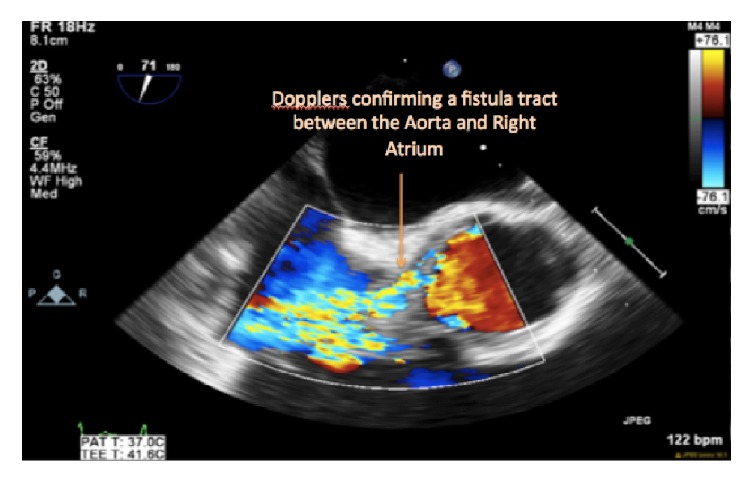
Axial image of the aortic root abscess with Doppler confirming the presence of a fistula between the aorta and the right atrium.

History was also significant for contact with a child who had streptococcal sore throat 3 weeks prior to presentation as the patient worked as a school teacher. At the outside hospital, the patient was found to have significant leukocytosis of 30,000, transaminitis (AST and ALT in the 2000s), and elevated creatinine. Initial chest X-ray revealed an enlarged cardiac silhouette but no infiltrates. A CT scan was performed, revealing a pericardial effusion and trace pleural effusion (Figures 1–6).

On arrival to our facility, muffled heart sounds and a soft diastolic murmur in the left lower sternal border were found on initial physical exam. A transthoracic echocardiogram revealed pericardial effusion and moderate mitral and tricuspid regurgitation. It also demonstrated vegetation on mitral valve.

Vancomycin, rifampin, and gentamicin were initially started as empirical endocarditis therapy but then changed to vancomycin and ceftriaxone after urine antigens and blood culture were positive for* Streptococcus pneumoniae*.

A formal transesophageal echocardiogram for better valvular evaluation was performed and confirmed mitral valve regurgitation with vegetation and tricuspid regurgitation with a large echo density. The most impressive aspect of the imaging was the presence of a fistula formation between the aorta and the right atrium. Cardiothoracic surgery was consulted and the patient underwent an emergent two-stage cardiac procedure. Initial surgery was performed three days after hospital admission and consisted in a median sternotomy with 500 cc to 750 cc of purulent pericardial drainage. Due to these findings, initial surgical plans of fistula and valvular repair and replacement were postponed and first surgical intervention became a primary washout without chest closure.

Postoperatively, the patient returned to the ICU and continued on vasopressors, extracorporeal membrane oxygenator (ECMO), and Intra-aortic Balloon Pump (IABP).

Two days later, the patient underwent a complex surgical repair with closure of the fistula, mitral, tricuspid valve repair, aortic replacement, and root enlargement. ECMO was continued postoperatively.

Despite the fact that all these measures were performed within five days of arrival, the patient continued to clinically deteriorate and died within a few days from persistent multiorgan failure.

## 2. Case Discussion

*Streptococcus pneumoniae* is an infrequent cause of acute endocarditis, accounting for less than 3% of all cases [1]. The classic presentation is an aortic valve infection in alcoholics, with concomitant pneumonia and meningitis, referred to as triad. Once endocardial infection is established, the course is typically aggressive and is associated with high mortality rates [2]. This is secondary to the release of toxins that cause rapid tissue destruction. Preceding infection with the H1N1 influenza virus is considered a risk factor for severe pulmonary infection. Our case represents a case of* Streptococcus pneumoniae* superinfection of an upper respiratory origin which resulted in severe cardiac complications consisting of mitral, tricuspid, and aortic valve endocarditis with valve destruction, fistula formation, and purulent pericardial effusion [3].

Accurate history, initial labs, imaging, and physical examination may be the first initial clues to a more serious and threatening complex process [4]. In our patient, physical exam revealed abnormal heart sounds consisting of a diastolic murmur and muffled heart sounds. Early recognition of* Streptococcus pneumoniae* sepsis and aggressive workup for complications such as an emergent transthoracic echocardiogram followed by a transesophageal echocardiogram is important. Empiric broad-spectrum antibiotics and aggressive hemodynamic support, followed by proper surgical intervention when warranted, are critical when bacterial superinfection is suspected as a delay may result in poorer outcomes [5].
